# Effect of automated identification of antimicrobial stewardship opportunities for suspected urinary tract infections

**DOI:** 10.1017/ash.2024.437

**Published:** 2024-10-03

**Authors:** Connor R. Deri, Rebekah W. Moehring, Nicholas A. Turner, Justin Spivey, Sonali D. Advani, Rebekah H. Wrenn, Michael E. Yarrington

**Affiliations:** 1 Department of Pharmacy, Duke University Medical Center, Durham, NC, USA; 2 Duke Center for Antimicrobial Stewardship and Infection Prevention, Durham, NC, USA; 3 Division of Infectious Diseases, Department of Medicine, Duke University Medical Center, Durham, NC, USA; 4 Department of Pharmacy, McLeod Health Seacoast, Little River, SC, USA

## Abstract

**Objective:**

We aimed to determine whether automated identification of antibiotic targeting suspected urinary tract infection (UTI) shortened the time to antimicrobial stewardship (AS) intervention.

**Design:**

Retrospective before-and-after study.

**Setting:**

Tertiary and quaternary care academic medical center.

**Patients:**

Emergency department (ED) or admitted adult patients meeting best practice alert (BPA) criteria during pre- and post-BPA periods.

**Methods:**

We developed a BPA to alert AS pharmacists of potential ASB triggered by the following criteria: ED or admitted status, antibiotic order with genitourinary indication, and a preceding urinalysis with ≤ 10 WBC/hpf. We evaluated the median time from antibiotic order to AS intervention and overall percent of UTI-related interventions among patients in pre-BPA (01/2020–12/2020) and post-BPA (04/15/2021–04/30/2022) periods.

**Results:**

774 antibiotic orders met inclusion criteria: 355 in the pre- and 419 in the post-BPA group. 43 (35 UTI-related) pre-BPA and 117 (94 UTI-related) post-BPA interventions were documented. The median time to intervention was 28 hours (IQR 18–65) in the pre-BPA group compared to 16 hours (IQR 2–34) in the post-BPA group (*P* < 0.01). Despite absent pyuria, there were six cases with gram-negative bacteremia presumably from a urinary source.

**Conclusions:**

Automated identification of antibiotics targeting UTI without pyuria on urinalysis reduced the time to stewardship intervention and increased the rate of UTI-specific interventions. Clinical decision support aided in the efficiency of AS review and syndrome-targeted impact, but cases still required AS clinical review.

## Introduction

To optimize antibiotic prescribing and prevent antibiotic-associated harm, antimicrobial stewardship programs (ASPs) comprehensively review antibiotic use and prescribing, evaluating the drug, dose, duration, and indication of therapy. However, ASPs are limited in personnel resources and many centralized, expert ASP teams cannot feasibly review all patients on antibiotics each day for prospective audit and feedback. Thus, stewards benefit from electronic tools to streamline workflows, prioritize cases that are more likely to get a successful stewardship intervention, and target areas of need for their hospital’s patients.^1^

Asymptomatic bacteriuria (ASB) is a common target for ASPs given its high prevalence in vulnerable patient populations, including the elderly, long-term care residents, and those with indwelling urinary catheters.^2,3^ The 2019 Infectious Diseases Society of America guidelines on the management of ASB recommend against screening and treating ASB except in patients who are pregnant or will undergo an endoscopic urologic procedure associated with mucosal trauma.^3^ Numerous studies have demonstrated a lack of benefit among ASB-treated patients with potential for harm; ASB treatment leads to an increased risk of antibiotic-associated harms, antibiotic resistance, and subsequent urinary tract infections (UTIs).^4–8^ Nevertheless, ASB treatment remains common. Thus, innovative antimicrobial stewardship (AS) initiatives are needed to combat unnecessary antibiotic use in ASB.^9–11^

Clinical decision support (CDS) systems (CDSS) are computer applications incorporated into the electronic health record (EHR) to provide alerts and/or guidance to frontline clinicians.^12^ The high negative predictive value (NPV) of absent pyuria for UTIs makes the urinalysis white blood cell (WBC) count result an appealing discrete electronic trigger for CDSS.^13^ However, to our knowledge, data are limited regarding utilizing CDSS to rapidly identify possible ASB-treated patients to optimize prescribing.

Therefore, we created an automated BPA for cases with new antibiotic orders, a genitourinary indication, and recent urinalysis showing the absence of pyuria (UA-BPA) directed to an AS pharmacist for review. This study aimed to evaluate the impact of the UA-BPA on time to AS intervention, the percent of AS interventions that were UTI-focused, and antibiotic length of therapy.

## Methods

### Study setting

This study was conducted at Duke University Hospital (DUH) in Durham, North Carolina (1,048 inpatient beds). DUH utilizes Epic© (2023 Epic Systems Corporation, Verona, Wisconsin; www.epic.com) as its EHR. The Antimicrobial Stewardship Evaluation Team (ASET) at DUH consists of three inpatient AS pharmacists and 1 physician FTE (shared among five adult ID physicians and 1 pediatric ID physician). ASP staffing and resource allocation did not change during the pre- and post-intervention periods. AS pharmacists and physicians routinely perform prospective audit and feedback weekdays on inpatients receiving antimicrobials but are unable to review all antibiotic patients in the facility (approximately 550 antibiotic exposed patients/day). AS pharmacists also routinely optimize therapy for patients with positive blood cultures, review customized BPAs for AS interventions (*eg,* bug-drug mismatch, de-escalation opportunities, etc), and perform allergy assessments with penicillin skin testing, as applicable. AS interventions are documented within the EHR.

Urinalysis and urine culture orders were separate at DUH without a reflexive urinalysis to urine culture order during the study period. Additional diagnostic stewardship interventions focused on UTIs were performed at DUH during the study period: (1.) creation of a urine culture order panel to assist in appropriate urine culture indications on 04/01/2021 and (2.) reporting of microscopic urinalysis bacteria and yeast results was discontinued on 8/30/2021.^14^

### Study design and participants

The UA-BPA identified ED or admitted patients with a new antibiotic order with an electronically associated “Genitourinary” indication and a preceding urinalysis (within 7 calendar days) with ≤ 5 white blood cell per high power field (WBC/hpf) (01/19/2021–04/14/2021). Antimicrobial indication is a required field within the EHR at the time of order entry. The WBC cutoff was modified to ≤ 10 WBC/hpf (04/15/2021–04/30/2022) to increase the sensitivity of the BPA. When criteria were met, a message was delivered to an ASP messaging pool which was reviewed on weekdays.

We analyzed antibiotic orders with associated AS interventions in a cohort of pre-BPA (01/01/2020–12/31/2020) and post-BPA intervention groups (04/15/2021–04/30/2022). Antibiotic orders were included for analysis if they met the following criteria: (1.) included “Genitourinary” indication, (2.) the associated patient had a preceding urinalysis with <10 WBC/hpf within 7 days (pre-BPA cohort) or the order triggered the CDS built on the same electronic criteria (post-BPA), and (3.) the associated patient’s age was ≥ 18 years. As antibiotic orders were the unit of analysis, multiple orders for an individual patient within the study period could be included. The initial post-BPA time frame using ≤ 5 WBC/hpf (01/19/2021–04/14/2021) was not included for analysis due to the brief time these criteria were used. This study was reviewed by the Institutional Review Board at DUH and determined exempt with a waiver for informed consent.

### Outcomes

The primary outcome was the time, in hours, from the antibiotic order entry (timestamp of electronic signature of ordering clinician) to any AS intervention occurring within 7 days. The seven-day period for interventions was chosen as this is a commonly selected duration of antibiotic therapy for many inpatient “Genitourinary” indications, and interventions beyond this duration may have been unrelated to the initial antibiotic order. A secondary outcome was the total percent of eligible patients that underwent intervention. Additional secondary outcomes among only intervened patients included type of AS intervention, the percent of AS interventions that were UTI-related, antibiotic length of therapy from criteria “trigger,” and development of bacteremia within 30 days.

### Data collection and statistical analysis

Patient demographics and BPA data were collected from Epic’s Clarity database. Electronic medication administration record (eMAR) data were obtained via the ASET operational database.^15^

We used descriptive statistics to report baseline demographic characteristics. Time to intervention and length of therapy data were nonparametric with rightward skew. To assess the incidence of antibiotic stewardship intervention, cumulative incidence plots were utilized for a time-to-event analysis, where the event was time to first antibiotic stewardship intervention. Patients were censored at discharge if this occurred within 7 days of CDS “trigger,” or censored at 7 days after antibiotic order entry. A Kaplan-Meier estimator was utilized for generation of cumulative incidence curves, with differences between curves assessed using the Log-rank test. The median time from antibiotic order entry to AS intervention within seven days and antibiotic length of therapy were compared pre- to post-BPA using the Mann Whitney U test. Rates of UTI-related interventions were compared with Fisher’s exact test. All statistical analyses were performed using Python v3.11.

## Results

### Primary outcome

**Figure 1.**
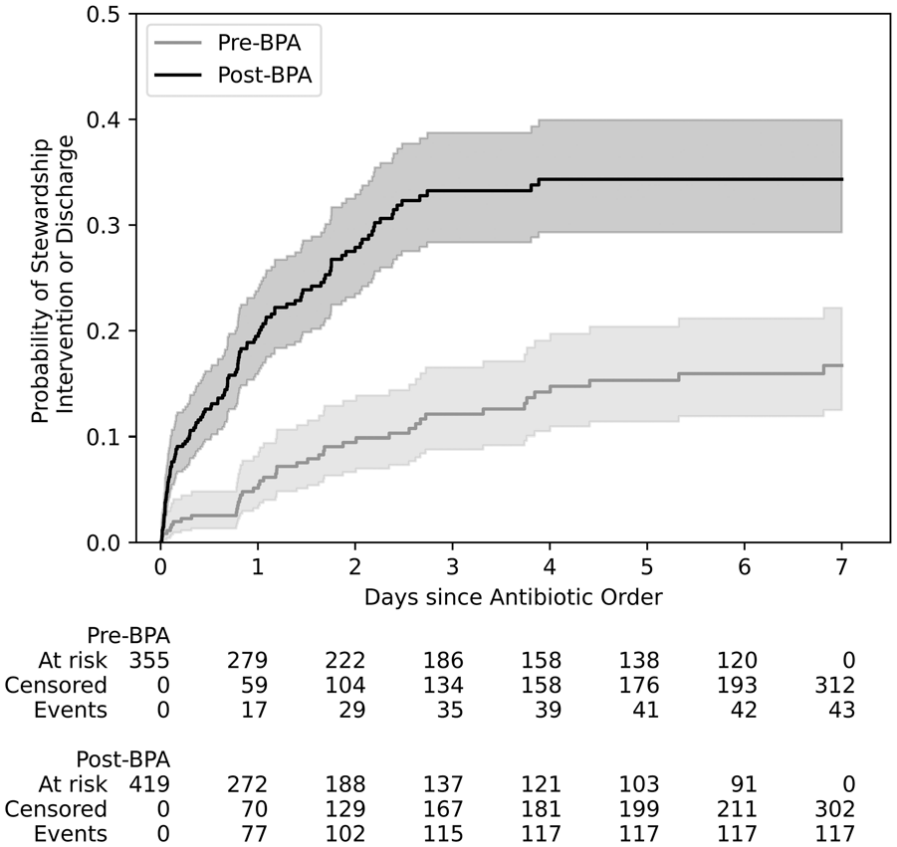
Time-to-event cumulative incidence plot.

774 antibiotic orders across 768 patients met criteria and were analyzed: 355 orders in the pre-BPA group and 419 in the post-BPA group. Six patients had an antibiotic order in both the pre- and post-BPA groups, while no patients had multiple orders within each group. Baseline characteristics were similar between cohorts (Table 1). The rate of AS interventions increased from 43/355 (12.1%) in the pre-BPA group to 117/419 (27.9%) in the post-BPA group [odds ratio (OR) = 0.36; 95% confidence interval (CI), 0.24–0.52]. The Kaplan-Meier log-rank test comparing time-to-event distributions indicated a statistically significant difference (log-rank p <0.005, Figure 1).

**Table 1.** Baseline patient characteristics among eligible antibiotic orders

Characteristics	Eligible pre-BPA (n = 355)	**Eligible post-BPA** **(n = 419)**
Median age, years (IQR)	65 (50–74)	64 (46–74)
Sex, male, n (%)	138 (38.9)	145 (34.6)
Race, n (%)		
White	208 (58.6)	237 (56.6)
Black	117 (33.0)	144 (34.4)
Other	30 (8.5)	38 (9.1)
Ethnicity, n (%)		
Hispanic	23 (6.5)	29 (6.9)
Not Hispanic	326 (91.8)	378 (90.2)
Not Reported/Declined	6 (1.7)	12 (2.9)
eGFR within 48 hours*, n (%)	320 (90.1)	303 (72.3)
Median eGFR (IQR)	69 (40–95) mL/min/1.73 m^2^	70 (45–92) mL/min/1.73 m^2^
Pregnant, n (%)	6 (1.7)	6 (1.4)
WBC within 48 hours*, n (%)	315 (88.7)	309 (73.7)
Median Serum WBC (IQR)	8.8 (6.4–12.8) × 10^9^/L	9.0 (6.3–12.2) × 10^9^/L
Urinary catheter, n (%)	96 (27.0)	85 (20.3)
Urinalysis, n (%)		
Positive nitrite	94 (26.5)	91 (21.7)
Urine culture in preceding 7 days*, n (%)	329 (92.7)	384 (91.6)
No growth	59 (16.6)	89 (23.2)
Mixed flora or	115 (32.4)	124 (32.3)
< 10,000 cfu/mL organisms		
Organism(s) identified	155 (43.7)	171 (44.5)
Urine culture organism, n (%)		
Enterobacterales	105 (29.6)	134 (32.0)
*Escherichia coli*	60 (16.9)	74 (17.7)
*Klebsiella pneumoniae* complex	30 (8.5)	39 (9.3)
*Proteus* spp.	8 (2.3)	8 (1.9)
Other	7 (2.0)	13 (3.1)
*Enterococcus* spp.	21 (5.9)	17 (4.1)
*Pseudomonas aeruginosa*	6 (1.7)	2 (0.5)
Other	23 (6.5)	18 (4.3)

BPA, best practice alert; IQR, interquartile range; eGFR, estimated glomerular filtration rate; WBC, white blood cell.

* Time from meeting clinical decision support criteria.

**Figure 2.**
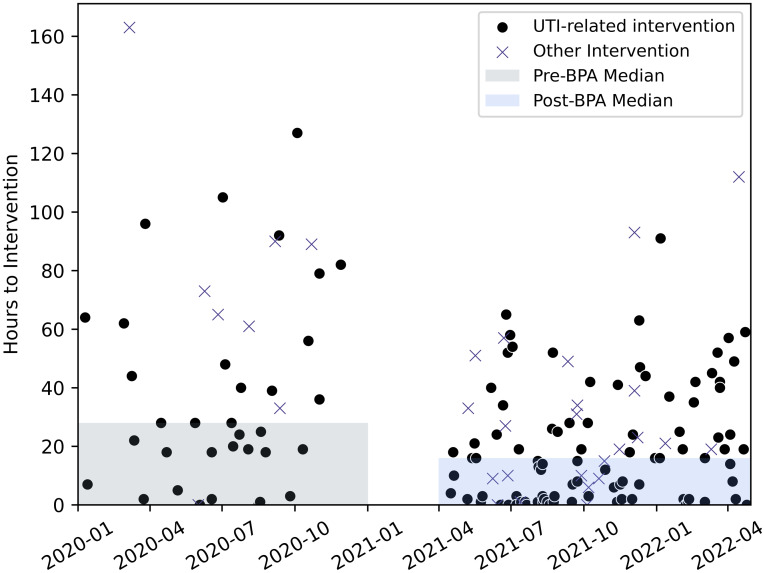
Time to AS intervention and UTI-related interventions.

The median time to AS intervention was significantly longer in the pre-BPA group compared to the post-BPA group [28 hours (IQR 18–65) vs 16 hours (IQR 2–34), *P* = < 0.01, 95% CI for the difference in medians, 5–36] (Figure 2).

### Secondary outcomes

**Figure 3.**
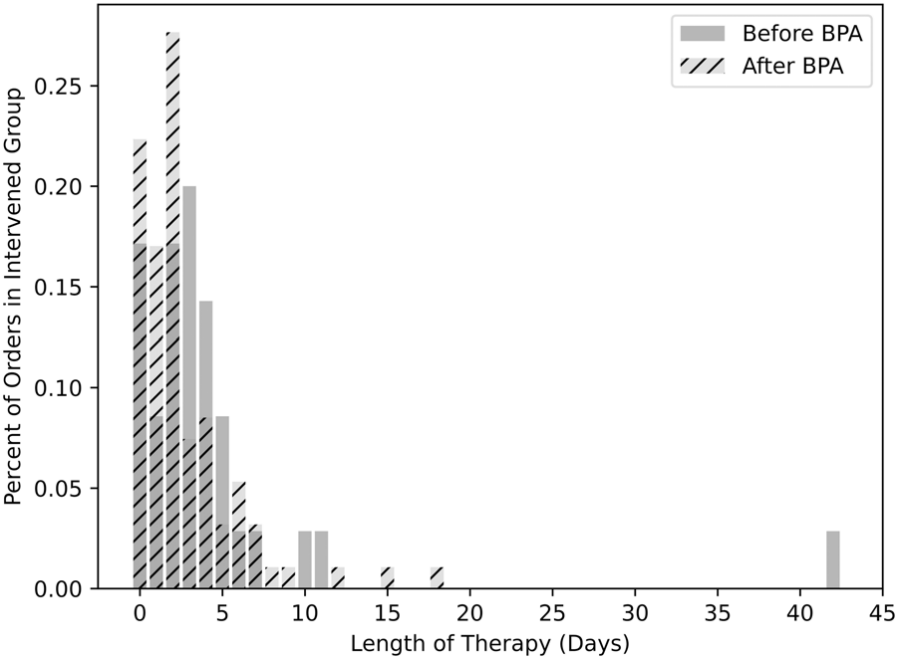
Length of therapy in intervened patients among pre- and post-BPA groups.

AS interventions were categorized by type of AS intervention. Interventions could have multiple AS intervention types (*eg,* de-escalation and IV to PO), if applicable. “Discontinue therapy” was the most common AS intervention type (n = 102, 63.4%) (Table 2). Furthermore, the pre-BPA group had a lower rate of UTI-related AS interventions compared to the post-BPA group (9.9% vs 22.4%, OR = 2.64, 95% CI, 1.74–4.02) (Figure 1). Among intervened patients, median antibiotic length of therapy was numerically longer in the pre-BPA group compared to post-BPA although did not meet statistical significance [4 days (IQR 2.5–6.0) vs 3 days (IQR 2.0–5.0), P = 0.052, 95% CI for the difference in medians, 0.0–2.0] (Figure 3). Bacteremia within 30 days of meeting criteria was compared between groups as a balancing measure. The pre-BPA group had 16/355 (4.5%) antibiotic orders with subsequent positive blood cultures within 30 days compared to 30/419 (7.2%) in the post-BPA group (OR = 1.63, 95% CI, 0.88–3.05). Among intervened patients, 7/43 (16.3%) in the pre-BPA group and 10/117 (8.5%) in the post-BPA group had positive blood cultures within 30 days of meeting criteria (OR = 0.48, 95% CI, 0.17–1.36). Most abnormal blood culture results were unrelated to a urinary source of infection and often consistent with blood culture contamination (Supplementary Material). However, we identified six patients in our cohort with gram-negative bacteremia [*Escherichia coli* (n = 3), *Proteus mirabilis* (n = 1), *Klebsiella pneumoniae* (n = 1), and a polymicrobial infection with *Escherichia coli* and *Klebsiella pneumoniae* (n = 1)] presumably from a urinary source within 3 days of urinalysis despite absence of pyuria. All six patients were in the post-BPA cohort. Two (33.3%) patients received an AS intervention at the time of bacteremia: (1.) change in therapy/escalation and (2.) antimicrobial de-escalation. Three (50%) of these patients had evidence of an obstructing ureteral calculus on computed tomography scan; five (83.3%) patients had at least 10 red blood cells/hpf and all six patients had signs or symptoms of infection (*eg,* fever, leukocytosis, dysuria, hematuria, and/or costovertebral angle tenderness).

**Table 2.** Antimicrobial stewardship intervention type by intervention period

AS intervention type(s)	Pre-BPA (n = 43) n, %	Post-BPA (n = 117) n, %	Total (n = 160) n, %
Change in Therapy or Escalation	2 (4.7)	3 (2.6)	5 (3.1)
De-escalation	0 (0)	6 (5.1)	6 (3.8)
De-escalation, IV to PO	4 (9.3)	2 (1.7)	6 (3.8)
Discontinue Therapy	25 (58.1)	77 (65.8)	102 (63.4)
Duration of Therapy	1 (2.3)	9 (7.7)	10 (6.3)
Informational	2 (4.7)	13 (11.1)	15 (9.4)
IV to PO	2 (4.7)	2 (1.7)	4 (2.5)
IV to PO, Duration of Therapy	1 (2.3)	1 (0.9)	2 (1.3)
Other	6 (14.0)	4 (3.4)	10 (6.3)

AS, antimicrobial stewardship; BPA, best practice alert; IV, intravenous; PO, oral.

## Discussion

CDSS implementation is an effective strategy to improve stewardship-related outcome measures, including decreased antibiotic consumption, narrowed spectrum of antibiotic usage, and faster time to stewardship intervention.^16–18^ Prior studies utilizing CDSS targeted at ASB have focused efforts on reducing urine testing in asymptomatic patients.^14,19^ To our knowledge, use of CDSS to rapidly identify possible ASB-treated patients for AS review has not previously been reported despite the fact that ASB is a common target for hospital ASPs. We created an automated UA-BPA targeted to AS pharmacists and assessed its impact on the time to AS intervention among 774 antibiotic orders. We observed a significant reduction in the time to AS intervention in the post-BPA group compared to the pre-BPA group. In addition, we observed a reduction in antibiotic length of therapy in the post-BPA group compared to the pre-BPA group, although this was not statistically significant. The automated UA-BPA message to AS personnel more efficiently identified high-yield patients for AS review and intervention as compared with the prior AS workflow.

Antibiotic review in the form of prospective audit and feedback (PAF) is a core strategy among ASPs. In a recent survey of ASPs, a large majority of ASPs (84%) reported performing PAF with only 64% of programs having information technology add-ons to assist with stewardship reviews.^1^ ASPs are often understaffed and under-resourced to perform robust PAF and other stewardship activities for all patients receiving antimicrobials.^20^ Given these resource limitations, CDSS play a key role in ASPs to improve workflow efficiencies and overall AS reach. Prior studies have demonstrated the benefit of CDSS within AS through reduced broad-spectrum antibiotic use,^21^ improved antibiotic susceptibilities,^22^ and improved clinical outcomes.^23^ However, data is limited in the use of CDSS to target ASB. Alternative ASB intervention strategies, including diagnostic stewardship and reflexive urine cultures, have also been successfully performed and described.^24^ There is likely more than one way to tackle inappropriate treatment of ASB and a multipronged approach may be warranted. The inappropriate treatment of ASB remains a target for ASPs across the country, including our institution. In this study, the UA-BPA enabled our AS team to rapidly identify patients for review and increase AS interventions, specifically targeting cases with possible ASB.

Among intervened patients, “discontinue therapy” was the most common recommendation from the AS team and we observed reduced antibiotic use in the post-BPA group compared to the pre-BPA group. Our study was not powered to detect a difference in median antibiotic duration; however, the observed values suggest statistical significance may be seen in a larger cohort.

Furthermore, the UA-BPA was purposefully designed to alert an AS pharmacist for review as opposed to a front-facing alert at the time of ordering by the clinician. Although the lack of pyuria has a strong NPV for ruling out UTI, it is not perfect for every clinical scenario. Though we considered making the BPA a front-facing alert, we wanted to test the criteria with an AS review back up to detect cases where criteria might falsely identify patients who have real infection rather than ASB. For instance, we observed six clinical cases of gram-negative bacteremia presumably from a urinary source despite absent pyuria. We also encountered additional cases with absent pyuria without bacteremia that necessitated antibiotic therapy (*eg,* obstructing stone with systemic symptoms, upper tract infection, and neutropenia). Thus, AS review provided a buffer between the alert and clinician and an opportunity for direct stewardship intervention with provider education. A front-facing alert based on these criteria might have reduced impact if ignored or, on the other hand, potentially lead to inappropriate antibiotic changes in the falsely identified cases as described above. Additionally, these cases provide a few examples where reflexive urine culturing using only pyuria would not have reflexed to a urine culture. Clinical correlation is imperative; symptomatic patients with obstructing ureteral stones likely still warrant urine cultures despite absent pyuria. Future investigations could determine the efficacy and safety of a front-facing UA-BPA on prescribing habits at the time antibiotic ordering without AS intervention.

This study had limitations. First, this was a retrospective, single-center before-and-after study with associated design limitations. Future studies might consider randomization techniques within the EHR to collect data on concurrent controls instead of a historical group. Additionally, this study was conducted at a large academic medical center with robust AS support and resources which may limit generalizability at other institutions depending on available EHR support, AS resources, and hospital size. We also removed bacteria and yeast reporting from urinalyses and created a urine culture order panel to facilitate appropriate urine culture ordering practices during the study period which may have decreased the number of ASB-treated patients, potentially biasing the results to the null hypothesis. Lastly, this study focused only on patients with absent pyuria and a genitourinary antibiotic indication. Thus, the UA-BPA alone would not capture all ASB-treated patients and should be used in conjunction with additional stewardship strategies. In addition to future studies to evaluate the BPA on front-facing alerts mentioned above, its impact in the outpatient setting may also show benefit as outpatient ASPs develop.

Automated identification of antibiotics targeting UTI with urinalysis showing absence of pyuria reduced the time to stewardship intervention and increased rate of UTI-specific interventions. The use of clinical decision support may aid in efficiency of AS review and syndrome-targeted AS impact.

## Supporting information

Deri et al. supplementary material Deri et al. supplementary material

## Data Availability

The data underlying this article will be shared on reasonable request to the corresponding author.

## Supplementary material

For supplementary material accompanying this paper visit http://doi.org/10.1017/ash.2024.437. click here to view supplementary material
