# Supplementary material for: Effect of automated identification of antimicrobial stewardship opportunities for suspected urinary tract infections
Source: Antimicrob Steward Healthc Epidemiol. 2024 Oct 3;4(1):e158. doi: 10.1017/ash.2024.437 (PMC11450662; doi:10.1017/ash.2024.437)
Supplement: Deri et al. supplementary material [file S2732494X24004376sup001.docx]

**Supplementary Material**

| **Blood Culture Pathogens (N = 50)** | **N (%)** | **Urine culture positive for the same organism (N, %)** |
| --- | --- | --- |
| **Typical Uropathogens** |  |  |
| *Escherichia coli* | 8 (16) | 5 (10)* |
| *Klebsiella pneumoniae* | 3 (6) | 2 (4) |
| *Proteus mirabilis* | 1 (2) | 1 (2) |
|  |  |  |
| **Common Skin Commensals/Blood Culture Contaminants** |  |  |
| *Staphylococcus epidermidis* | 12 (24) | 0 (0) |
| *Staphylococcus capitis* | 3 (6) | 0 (0) |
| *Bacillus spp* | 3 (6) | 0 (0) |
| *Micrococcus luteus* | 2 (4) | 0 (0) |
| *Staphylococcus hominis* | 2 (4) | 0 (0) |
| *Staphylococcus haemolyticus* | 1 (2) | 0 (0) |
| *Streptococcus mitis/oralis* | 1 (2) | 0 (0) |
|  |  |  |
| **Other Pathogens** |  |  |
| Methicillin-resistant *Staphylococcus aureus* | 3 (6) | 0 (0) |
| *Bacteroides thetaiotaomicron* | 2 (4) | 0 (0) |
| *Enterococcus faecium* | 2 (4) | 0 (0) |
| *Clostridium spp* | 1 (2) | 0 (0) |
| *Enterobacter cloacae complex* | 1 (2) | 0 (0) |
| *Moraxella osloensis* | 1 (2) | 0 (0) |
| *Neisseria gonorrhoeae* | 1 (2) | 0 (0) |
| *Pantoea agglomerans* | 1 (2) | 0 (0) |
| *Prevotella denticola* | 1 (2) | 0 (0) |
| *Pseudomonas aeruginosa* | 1 (2) | 0 (0) |

**Supplementary Table 1. Positive Blood Cultures within 30 Days of Antibiotic Order**

*4 out of 5 (80%) positive blood cultures with *E.* *coli* occurred within 3 days of BPA ‘trigger’
